# Study on the Behavioural Assessment of the Dysexecutive Syndrome (BADS) performance in healthy individuals, Mild Cognitive Impairment and Alzheimer’s disease: A preliminary study

**DOI:** 10.1590/S1980-57642009DN30200006

**Published:** 2009

**Authors:** Cristiane Garcia da Costa Armentano, Cláudia Sellitto Porto, Sonia Maria Dozzi Brucki, Ricardo Nitrini

**Affiliations:** 1 Post Graduate Student, Neuropsychologist, Behavioral and Cognitive Neurology Unit, Department of Neurology, University of São Paulo School of Medicine, São Paulo, SP, Brazil.; 2 PhD, Behavioral and Cognitive Neurology Unit, Department of Neurology, University of São Paulo School of Medicine, São Paulo, SP, Brazil.; 3 MD, Behavioral and Cognitive Neurology Unit, Department of Neurology, University of São Paulo School of Medicine, São Paulo, SP, Brazil.; 4 MD, PhD, Behavioral and Cognitive Neurology Unit, Department of Neurology, University of São Paulo School of Medicine and Cognitive Disorders Reference Center (CEREDIC) of Hospital das Clínicas of the University of São Paulo School of Medicine, São Paulo, SP, Brazil.

**Keywords:** Mild cognitive impairment, Alzheimer’s disease, executive functions, BADS, neuropsychological tests

## Abstract

Executive deficits as well as deficits in episodic memory characterize the initial phases of Alzheimer Disease (AD) and are clinically correlated to neuropsychiatric symptoms and functional loss. Patients with Mild Cognitive Impairment present more problems as to inhibitory response control, switching and cognitive flexibility.

**Objective:**

To compare performance on the BADS with performance on other executive functional tests among patients with mild Alzheimer’s disease, Amnestic Mild Cognitive Impairment (aMCI) to performance of control individuals and to examine discriminative capacity of BADS among these groups.

**Methods:**

The BADS was performed by 35 healthy controls, 13 patients with aMCI, and 16 mild probable AD patients. Besides performing the BADS, subjects underwent neuropsychological evaluation which comprised: the Dementia Rating Scale (DRS), verbal fluency by phonemic categories (F.A.S) and Concentrated Attention Test (CA).

**Results:**

There were no differences among groups by educational level, but performance differed for age (p<0.01). No difference between healthy controls and aMCI patients was found on total scores or subitems of the BADS. A significant difference was observed between aMCI and AD patients (p<0.05) and between controls and AD patients (p<0.05) on total and standard scores.

**Conclusions:**

Performance on the BADS differed between healthy individuals and mild AD patients. The BADS proved to be a sensitive method for discriminating AD from aMCI.

## Abstract (pt)

Déficits executivos conjuntamente aos déficits de memória episódica caracterizam as fases iniciais da doença Alzheimer (DA) e se correlacionam clinicamente com sintomas neuropsiquiátricos e com prejuízo funcional. Pacientes com Comprometimento Cognitivo Leve apresentam mais problemas com controle inibitório de respostas, alternância e flexibilidade cognitiva.

**Objetivo:**

Comparar o desempenho na BADS e outros testes de funções executivas entre pacientes com demência Alzheimer de intensidade leve, comprometimento cognitivo leve tipo amnéstico (CCLa) e indivíduos controles e verificar a sensibilidade da BADS para discriminar CCL tipo amnéstico de DA.

**Métodos:**

A BADS foi administrada a 35 indivíduos saudáveis, 13 pacientes com CCL amnéstico, e 16 pacientes com DA provável de intensidade leve. A avaliação consistiu da BADS e por avaliação neuropsicológica composta por: Escala de Avaliação de Demência(DRS), fluência verbal por categorias fonêmicas (F.A.S.) e o Teste de Atenção Concentrada (AC).

**Resultados:**

Não houve diferença estatisticamente significativa entre os grupos quanto à escolaridade, mas houve diferença quanto à idade (p<0,01). Não houve diferença estatisticamente significativa entre indivíduos controles e pacientes com CCLa nos subtestes e nos escores totais da BADS. Houve diferença significativa entre pacientes com CCLa e pacientes com DA (p<0,05) e entre indivíduos controles e pacientes com DA (p<0,05) nos escores totais padronizados.

**Conclusões:**

Foram encontradas diferenças no desempenho na BADS entre indivíduos saudáveis e o grupo de pacientes com DA. A BADS mostrou-se sensível para discriminar diferenças de desempenho entre DA e CCLa.

Alzheimer’s disease (AD) is the most common cause of dementia representing more than 50% of diagnosed cases in the 65 years and older age range and the most frequent dementia diagnosis in our practice.^1^

At the initial stages of AD, the remote memory remains relatively preserved, however there is recent memory impairment which interferes in functional activities of daily living. Additionally, the cognitive evaluation has been shown to involve other functions such as: attention, naming, reasoning and visual-spatial skills.^2^ The executive deficits also characterize the initial phases of AD^3-5^ and are clinically correlated to neuropsychiatric symptoms^6^ and functional damage.^6,7^

In the literature, the concept of *Mild Cognitive Impairment (MCI)*, proposed by Petersen et al.,^8,9^ has been used in several studies to designate an intermediate state between normality and dementia suggesting that clinically, there is a risk of a prodomal state for Alzheimer’s disease^9-12^ with a conversion rate of 12% for every year of age.^9^

Traykov et al.,^5^ compared patients diagnosed with MCI to control patients and concluded that there was impairment in the performance of episodic memory, response inhibitory control, switching and cognitive flexibility which involved several aspects of executive functions. They suggested that MCI may be identified by using more comprehensive procedures rather than solely memory evaluation.

However, Rozzini et al.,^13^ followed patients diagnosed with Amnestic Mild Cognitive Impairment (aMCI) for a year to verify the risk factors for conversion into AD. During follow-up, patients who became demented had a worsening in executive functions (inhibitory control and cognitive flexibility) and in functional daily activities, while memory function did not deteriorate. Memory function performance in the long run was not associated to conversion into AD.

Patients with MCI demonstrated subtle alterations in functional daily activities that were associated to executive dysfunction.^14^

During the aging process, both normal and pathologic executive functions tend to be impaired. A possible hypothesis for such decline is the natural physiological frontal lobe deterioration.^15^ According to Jacobson, Delis, Bondi and Salmon,^16^ executive decline may anticipate the onset of a dementia by seven to ten years.

In this sense, the executive skills should be targeted as important initial markers of pathological processes.

It is necessary to study the neuropsychological profiles that help form clinical markers for elderly evaluation, especially in functions such as executive skills.

The Behavioural Assessment of the Dysexecutive Syndrome (BADS)^17^ is a battery of tests used to evaluate problems that arise during daily living activities due to Dysexecutive Syndrome (DES). It is composed of thirteen tasks grouped into six subtests and two questionnaires, and is designed to estimate executive functional aspects such as: planning disorders, inhibitory control, problem solving, temporal judgment and behavioural alterations.

The objectives of this study was to compare the performance of the BADS against other executive functional tests among patients with mild Alzheimer’s disease, Amnestic Mild Cognitive Impairment (aMCI) and control individuals in order to verify the sensitivity of the BADS to discriminate amnestic-type MCI from AD.

## Methods

### Casuistic

We evaluated 64 subjects distributed into three groups: control (35), patients with aMCI (13) and patients with Alzheimer disease (16).

The diagnosis among the aMCI group was based on the criteria of Petersen et al.^8,9^ whereas for the probable AD diagnosis, the criteria from the Institute of Neurological Diseases and Communicative Disorders and Stroke-Alzheimer’s Disease and Related Disorders Association (NINCDS-ADRDA) were adopted.^18^ The diagnosis of mild dementia was reached according to the criteria from the Manual of Diagnosis and Statistics of Mental Disorders, 3^rd^ revised Edition, (DSM-III-R).^19^ All AD patients had been regularly taking anticholinesterasic medication and anti-depressive drugs for at least two months. All patients were recruited from the Cognitive and Behavioural Neurology Group (GNCC) and the Reference Center of Cognitive Disorders (CEREDIC) of the Hospital das Clínicas of the University of São Paulo School of Medicine (HC-FMUSP).

All patients were 50 years old or above and had an educational level of at least 4 years.

– **Inclusion criteria:** Presence of an informant able to supply consistent data; the participants had to undergo a neurological examination, additional tests and neuropsychological assessment.
– **Exclusion criteria:** Moderate or severe dementia. Dementia of a different etiology. Presence of other conditions that compromise cognition such as: non-treated depression or other psychiatric diseases, use of psychotropic drugs, history of alcohol or chemical addiction, decompensated systemic disease, visual and/or hearing disorders without correction.

The control group was formed by volunteers from the community or patients’ spouses, without subjective memory complaints and who were independent for daily activities. All members of the control group were 50 years old or over and had an educational level of at least 4 years.

– **Inclusion criteria:** Score on the Mini-Mental State Mini-Examination (MMSE) greater than or equal to the mean for schooling according to Brucki et al.;^20^ minimum delayed recall score of five on the Brief Cognitive Screening Battery (BCSB);^21,22^ cut-off score of 3.4 on the *Informant Questionnaire on Cognitive Decline in the Elderly* (IQCODE);^23,24^ score of ≤2 on Basic Functional Assessment by means of the Functional Activities Questionnaire (FAQ);^21,25^ score of ≤22 points on the Subjective Memory Complaints Questionnaire (MAC-Q).^26,27^
– **Exclusion criteria:** Neurological disease; alcohol addiction, depression, other psychiatric disorders, non-corrected visual or hearing disorders, motor impairment and individuals under medication that may affect cognitive functions. Chronic disorders were not exclusion criteria if under control, such as arterial hypertension, diabetes mellitus and heart diseases.

### Neuropsychological evaluation

Neuropsychological Evaluation included:

- Behavioral Assessment of the Dysexecutive Syndrome – BADS.^17^
- Dementia Rating Scale (DRS)^28-30^ maximum score of 144.
- Verbal Fluency for phonemic categories (F.A.S.).^31^
- Concentrated Attention Test (CA).^32^

Behavioral Assessment of the Dysexecutive Syndrome – BADS^17^ is composed of six sub-tests, with a maximum score of 24 points. An overall Total Score (TS) for the whole battery is obtained by adding together the individual score for each subtest. The Total Score for each subject is converted into a Standard Score (SS) with a mean of 100 and a standard deviation of 15, and into an Age Standard Score (ASS). An overall classification is obtained: impaired, borderline, low average, average, high aver­age, superior, and very superior.

This battery of tests evaluates executive functions such as: inhibitory control and monitor behavior, planning, pri­orities, problem solving, and cognitive flexibility.

– ***Rule Shift Cards test:*** There are 21 cards. Patients in the first rule must say “yes” for red cards and “no” for black ones and in the second rule must say “yes” if two sequential cards are the same color and “no” if colors were different. This rule, typed on a card, is left in full view (for the patient) throughout, to reduce memory constraints. Maximum score of 4.
– ***Action Program test:*** The subject is presented with a rectangular stand into one end of which, a large trans­parent beaker is placed with a removable lid having a small central hole in it. A thin transparent tube at the bottom of which is a small piece of cork is placed into the other end of the stand. The beaker is two thirds full of water. To the left of the stand, a metal rod is placed (roughly- L- shaped) which is not long enough to reach the cork, and a small screw top container on its side, with its top unscrewed and lying beside it. Subjects are asked to get the cork out of the tube using any of the objects in front of them but without lifting up the stand, the tube or the beaker and without touching the lid with their fingers. Maximum score of 4.
– ***Key Search test:*** Subjects are presented with an A4-sized piece of paper with a 100 mm square in the middle and a small black dot 50 mm below it. The subjects are told to imagine that the square is a large field in which they have lost their keys. They are asked to draw a line, starting on the black dot, to show where they would walk to search the field to make absolutely certain that they would find their keys. Maximum score of 4.
– ***Temporal Judgment test:*** 4 questions about common events which take from a few seconds to several years and subjects are asked to guess the time of each event (seconds, minutes and, years). Maximum score of 4.
– ***Zoo Map test:*** Subjects are required to show how they would visit a series of designated locations on a map of a zoo. However, when planning the route certain rules must be obeyed (in our research these rules were left in front of participant as a memory cue). The map and rules have been constructed so that there are only four variations on a route that can be followed in order that none of the rules of the test are infringed. There are two trials. Maximum score of 4.
– ***Adapted version of The Modified Six Elements test:*** The subject has ten minutes to complete three different tasks (geometrical figures to be copied, picture naming and arithmetic), divided into two parts (A and B). The subjects are required to attempt at least something from each of the six sub tasks within the time allotted. They are not allowed to do two parts of the same task con­secutively (e.g. naming and arithmetic of part B), and are advised to do a little of some part. The rules were left in front of subjects. This test measures how well subjects organize themselves. Maximum score of 4.

### Behavioral evaluation

The Dysexecutive Questionnaire (DEX) of the BADS, is not used in the calculation of the profile score for the bat­tery. This comprises a 20-item questionnaire construct­ed in order to examine the range of problems associ­ated with the Dysexecutive syndrome. Two score forms were used: self-ratings performed by patient, and other ratings form completed by caregiver, with a maximum score of 80 points.

All the participants signed an informed consent and this study was approved by the Research Ethics Committee of the Hospital das Clínicas of the University of São Paulo School of Medicine.

### Statistical analysis

Calculations were performed to evaluate associations between the variables using the Mann-Whitney test. For continuous variables, and more than two variables, the Kruskal-Wallis test was employed with comparison of Dunn. After Bonferroni correction the level of significance adopted was p=0.0167. All the statistical analyses were carried out using the Statistical Package software for the Social Sciences, version 10.0 (SPSS).

## Results

There was no statistically significant difference between control and patient groups for Educational level (p=0.15) and gender (p=0.13), but a difference for age (p <0.01) was observed. The descriptive data of the sample are shown in Table 1.

**Table 1:** Sample descriptive data.

Group	N	Age	Gender	Schooling	MMSE*	FAQ^+^	IQCODE^++^
Controls	35	66.7 (7.9)	24/11	8.8 (4.7)	28.0 (1.5)	0	3.0 (0)
MCI	13	73.5 (7.3)	5/8	9.5 (4.9)	27.1 (1.4)	2.1 (1.4)	3.2 (0)
AD	16	78.8 (4.6)	11/5	6.5 (4.0)	23.6 (3.3)	13.1 (7.1)	3.9 (0.6)

Mean values and standard deviation.

* MMSE, Mini-Mental State Examination;

+ FAQ, Basic Functional Assessment by means of a Functional Activities Questionnaire;

++ IQCODE, Informant Questionnaire on Cognitive Decline in the Elderly.

Table 2 shows a comparison of the performance among patients and control subjects on neuropsychological assessments and the BADS.

**Table 2:** Comparison among control subjects, aMCI patients, and AD patients on BADS, DRS, F.A.S. and CA.

	Controls Mean (SD)	aMCI patients Mean (SD)	AD patients Mean (SD)
BADS (TS)	14.6 (3.1)^+^	13.0 (2.6)*	8.6 (3.8)*^+^
BADS (SS)	83.4 (15.4)^+^	75.3 (12.3)*	53.3 (19.5)*^+^
BADS (ASS)	87.3 (14.6)^+^	83.1 (13.1)*	61.1 (20.2)*^+^
DRS (TS)	136.6 (4.0)	129.6 (8.4)	112.1(12.8)
F.A.S.	32.0 (8.3)^+^	31.9 (8.1)	24.8 (9.0)^+^
CA	49.7 (16.8)^+^	37.2 (21.7)	26.6 (17.1)^+^

BADS, Behavioural Assessment of Dysexecutive Syndrome; TS, total score; SS, standard score; ASS, age standard score; F.A.S., verbal fluency by verbal categories; CA, concentrated attention; Kruskal-Wallis test:

* Comparison between MCI and AD patients (p<0.01);

+ Comparison between control subjects and AD patients (p<0.01).

The concentrated attention test showed statistically significant difference between control and AD groups (p<0.001). The aMCI group showed no difference compared to the AD group (p=0.253) or the control group (p=0.046).

The aMCI group showed no difference compared to controls on all subtests of the BADS (p>0.01) for Total Score (p>0.01), Stan­dard Score (p>0.01) and Age Standard Score (p>0.01).

The rule shift cards subtest (p<0.01), the zoo map test (p<0.01) and the modified six elements subtest (p<0.01) showed statistically significant difference in differentiating control subjects from AD patients.

## Discussion

During the aging process, deficits in episodic memory and executive control of neuropsychological tasks occur, especially associated to reduced information processing, attentional, inhibitory processes and cognitive flexibility.^13,33,34^ These alterations may be explained by the aging hypotheses of the frontal system.^35,36^ In our sample, control subjects and patient groups differed according to age. The control group had a lower mean age than both the aMCI and AD groups. However, there are considerable differences concerning age and executive functions. Salthouse and Ferrer-Caja^37^ reported that several studies have been conducted to investigate the executive performance throughout life and have observed a reduction in performance as age progresses. Wecker, Hallom and Delis^38^ noted that some studies show a decline in executive functions as age increases, yet others found no significant changes in this relationship. In a study carried out by Souza et al.,^39^ 61 adults with ages ranging from 19 to 70 years were evaluated. They used a minimum of 7 years education with the Wisconsin Cards Classification and Tower of London tests. The results demonstrated that executive performance tended to decline with age and was facilitated by educational level. Our control patients had a mean educational level of 9.0±4.8 years as did those in the study by Souza et al.,^39^ this fact may have been a facilitator offsetting executive decline by age. However, the influence of this variable on our study has yet to be verified.

Clinical observations in patients with Alzheimer disease regarding daily living situations suggest that there are problems maintaining attention at the initial stages of the disease.^40^ We observed differences in performance on the concentrated attention test between control groups and patients with AD, where AD patients performed worse than controls. Our findings are consistent with several studies suggesting that investigation of attentional performance may reveal possible damage in terms of memory and future learning capabilities.^41^ Moreover, such investigation can also detect early alterations in these functions in Alzheimer disease.^42-45^ No significant differences were found in comparisons among aMCI, AD and control groups. Our patients with aMCI showed relatively preserved performance for concentrated attention corroborating results of Traycov et al.,^44^ and de Perry et al.,^45^ who considered concentrated and divided attention to be preserved in MCI.

The verbal fluency test has been widely employed in executive function assessment.^31^ In our research the total number of words evoked in the phonological verbal fluency test differed significantly among groups. The *post-hoc* analysis revealed that the control group was significantly different from the group with AD patients while the aMCI did not differ to control subjects or AD patients.

Differentiating cognitive alterations typical of aging from initial manifestations of dementia states remains challenging. MCI has therefore been a focus of attention in research involving pre-clinical AD manifestations.^46^ Although MCI prognosis is heterogeneous, there seems to be a consensus that subjects with an MCI diagnosis are at a higher risk of progressing to dementia.

A recent study by Traykov et al.^44^ suggested that patients with MCI may be identified by using more detailed procedures rather than memory assessment alone and that patients with MCI present impairment to several aspects of executive functions. Canali, Brucki and Bueno^47^ published the preliminary results from a study involving the BADS to verify executive function performance in healthy elderly patients with probable AD at initial phases, and the applicability of this ecological assessment in Brazil. They found differences among groups and confirmed the efficacy of the BADS in detecting executive deficits. Our results confirmed these previous findings with differences observed among AD patients and controls. aMCI and AD patient groups showed no difference for age, but differed on the BADS scores. No difference was apparent among controls and aMCI patients. This may mean that the BADS is not the most suitable instrument to differentiate aMCI from control subjects either because the number of patients is small or it may lack sensitivity. We should also take into consideration that our aMCI group was older than controls which could have led to a bias in favor of detecting executive function impairment. We found no similar reports using BADS to evaluate executive functions in patients with MCI.

There was no difference in 3 sub items of the BADS (action programming, key search, temporal judgment). However, three other subtests of the BADS suggested greater diagnostic sensitivity (rule shift cards, zoo map test, modified six elements subtest). Our results are consistent with other studies that found difficulties in planning and demonstrated a decline in performance on the six modified elements subtest^47-49^ and zoo map test.^50^ A difference was also seen on total scores, stan­dard, standard by age and overall classification by age. We utilized Bonferroni correction to maintain the level of significance. After correction, patients with aMCI did not present significant differences compared to controls. Nevertheless, we found statistically significant differences between control subjects, AD patients and aMCI patients compared with the AD group. Our findings suggest that the BADS is sensitive for discriminating differences in performance between aMCI and AD, suggesting that executive functions may be used as an assessment marker to define what kind of patient with aMCI may convert to AD.
